# scMEB: a fast and clustering-independent method for detecting differentially expressed genes in single-cell RNA-seq data

**DOI:** 10.1186/s12864-023-09374-6

**Published:** 2023-05-25

**Authors:** Jiadi Zhu, Youlong Yang

**Affiliations:** School of Mathematics and Statistics, Xidian University, Xi’an, China

**Keywords:** Minimum enclosing ball, Differentially expressed genes, Single-cell RNA-seq data

## Abstract

**Background:**

Cell clustering is a prerequisite for identifying differentially expressed genes (DEGs) in single-cell RNA sequencing (scRNA-seq) data. Obtaining a perfect clustering result is of central importance for subsequent analyses, but not easy. Additionally, the increase in cell throughput due to the advancement of scRNA-seq protocols exacerbates many computational issues, especially regarding method runtime. To address these difficulties, a new, accurate, and fast method for detecting DEGs in scRNA-seq data is needed.

**Results:**

Here, we propose single-cell minimum enclosing ball (scMEB), a novel and fast method for detecting single-cell DEGs without prior cell clustering results. The proposed method utilizes a small part of known non-DEGs (stably expressed genes) to build a minimum enclosing ball and defines the DEGs based on the distance of a mapped gene to the center of the hypersphere in a feature space.

**Conclusions:**

We compared scMEB to two different approaches that could be used to identify DEGs without cell clustering. The investigation of 11 real datasets revealed that scMEB outperformed rival methods in terms of cell clustering, predicting genes with biological functions, and identifying marker genes. Moreover, scMEB was much faster than the other methods, making it particularly effective for finding DEGs in high-throughput scRNA-seq data. We have developed a package scMEB for the proposed method, which could be available at https://github.com/FocusPaka/scMEB.

**Supplementary Information:**

The online version contains supplementary material available at 10.1186/s12864-023-09374-6.

## Background

Advances in single-cell RNA sequencing (scRNA-seq) have made it possible to assess gene expression and cell state at high resolution [1–3]. Conventional bulk RNA-seq investigates the transcriptomes by average cellular expression, which may ignore the differences between individual cells. Developed scRNA-seq technologies have provided opportunities to capture expressions of genes at the cellular level and further elucidated the heterogeneity between different cells [4, 5]. However, the identification of differentially expressed genes (DEGs) across cells is still a challenging problem.

Multiple tools have been developed for bulk or scRNA-seq differential expression analysis. Parametric methods including edgeR [6], DESeq2 [7], BPSC [8], and DEsingle [9], which identify DEGs based on different distribution assumptions. Non-parametric methods, such as the Kolmogorov–Smirnov test, NODES [10], the Wilcoxon rank-sum test [11] and the likelihood ratio test, which detect DEGs directly. Soneson and Robinson [12] noted that some methods designed for bulk RNA-seq data differential expression analysis could be used for scRNA-seq but only if genes with low expression are prefiltered. One commonality these methods share is to identify DEGs between two experimental conditions. However, in some cases, the true labels of cells are implicit or unknown, and there may be two or more potential types of cells.

To overcome the above issues, a straightforward solution is to carry out unsupervised clustering first and then perform the differential expression analysis with the above tools by comparing one cluster to the rest of the clusters. In this way, clustering results affect the downstream analysis enormously. Choosing an appropriate clustering method and setting the parameters to get the perfect clustering result remains an issue. We clustered 11 real datasets by graph-based methods in the scran [13] package with different parameter settings and found the difference in the number of types between the clustering results and the reference labels (Supplementary file 1 Table S1). Considering the drawbacks, some methods have been proposed to identify DEGs without reference clustering results. Representative methods include singleCellHaystack [14] and MarcoPolo [15]. singleCellHaystack employs Kullback–Leibler divergence to find genes with expressions that are non-uniformly distributed in a low-dimensional space. MarcoPolo assumes scRNA-seq data are bimodal and fit each gene with a Poisson mixture model, and it sorts out DEGs using three criteria. Different from other methods that obtain the DEGs only under two conditions, the above two methods directly detect DEGs without clustering results, which improves the calculation accuracy and efficiency enormously. However, we find that the problem of runtime still exists for these two methods when dealing with sizable real datasets, especially for the MarcoPolo method.

**Fig. 1:**
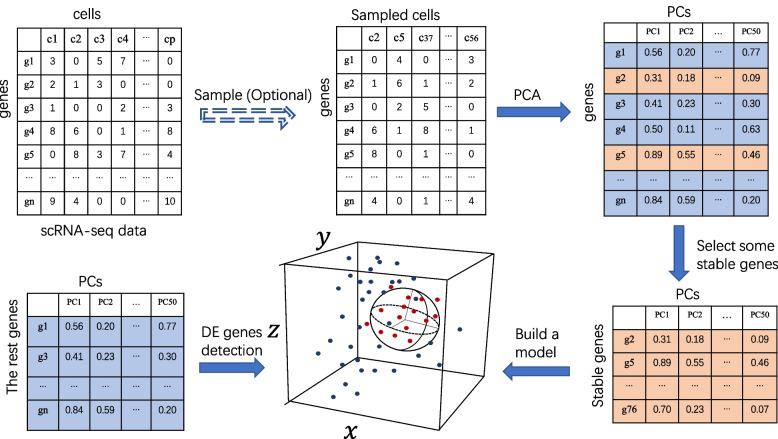
Overview of the scMEB workflow. Randomly sampling some important cells before PCA is optional, and the purpose is to speed up the calculation. The first 50 PCs could be obtained from the sampled/unsampled cells. A small part of the stable genes was used to build the model. A sphere that encloses the training data in the feature space was found. The remaining genes were divided into DEGs or non-DEGs according to whether they were outside or inside the enclosing ball, respectively

Here, we present single-cell minimum enclosing ball (scMEB), a novel and fast method for detecting single-cell DEGs. It extends the existing bulk RNA-seq differential expression analysis method scaling-free minimum enclosing ball (SFMEB) [16] to scRNA-seq data. It assumes that a small part of non-DEGs is known and builds a minimum enclosing ball based on it, and the genes outside the ball are regarded as DEGs. This method does not require data normalization beforehand and could be easily applied to scRNA-seq datasets without labelling cells. Because the method only uses parts of genes to build the model, it has the advantages of simple formulation and quick calculation speed. The calculation efficiency of the method could be further improved by using principal components (PCs) as inputs. Figure 1 shows the workflow of scMEB for detecting DEGs, and the details of the proposed methods are shown in the Methods section.

Compared with the SFMEB method, scMEB is advanced in the wider scope of application, the improvement of computation accuracy and speeding up the runtime of identifying DEGs. First, the proposed scMEB method is developed to identify DEGs in subsets of scRNA-seq data with many types of cells or scRNA-seq data with unknown labels. However, the SFMEB method only considers the DEG detection of bulk RNA-seq data. The purpose of SFMEB is to detect DEGs under two biological conditions or two different species. In reality, the prediction of differential expression patterns in single-cell RNA-seq data is more complex than in RNA-seq data. One of the major problems is that the evaluation of the cluster number is difficult because of the high dimensionality of scRNA-seq data. Besides, for scRNA-seq data with low abundance populations, some genes are expressed differentially in a sub-population but not detected in a larger cluster [14], which may lead to false negatives. Second, the SFMEB only considers building the model with the raw read counts, however, scMEB could transform the original expressions, and build the model with the transformed data. In our analysis, we incorporate PCA in the model and build the model with the first PCs. This difference not only improves the scMEB computation accuracy enormously but also speeds up the calculation greatly, especially for a sizable dataset.

## Results

### Descriptions of real data analysis

In this section, we compared the performance of scMEB with MarcoPolo and the four modes of singleCellHaystack. All three methods were developed for data that cell clusters were not obvious to detect DEGs without prior clustering. Compared with other methods under two conditions, these three methods were faster and more accurate for DEG detection [14, 15]. scMEB needs some non-DEGs to build the model; however, the ground truth of the genes is unknown. To address this issue, we utilized stably expressed genes (SEGs) [17] to replace non-DEGs to build the model. SEGs refer to a subset of genes in single cells that are stably expressed in different cells and tissues, and the function is identical to the housekeeping genes in RNA-seq data [17]. For subsequent analysis, we directly used lists of 1,076 human and 916 mouse SEGs downloaded from https://sydneybiox.github.io/scMerge/reference/scSEGIndex.html to build the model. In addition, we could use the scSEGIndex [17] method to get the SEGs of any species and the R package scMerge [18] as the calculation framework.

### Distribution comparison of the identified DEGs

We analyzed the distributions of the most significant 500 DEGs obtained by these three methods. The three methods measure the significance level of a gene’s potential as a DEG in different ways. The four modes of singleCellHaystack return estimated *p*-values for each gene. The smaller the *p*-value, the more significant this gene is. MarcoPolo returns a rank for each gene based on three criteria; the higher the ranking, the more significant. Finally, scMEB uses the signed distance between the point and the sphere of the hypersphere in feature space to measure the significance level of a gene’s potential as a DEG, and the larger the distance is, the more likely it is that the gene is a DEG.

**Fig. 2:**
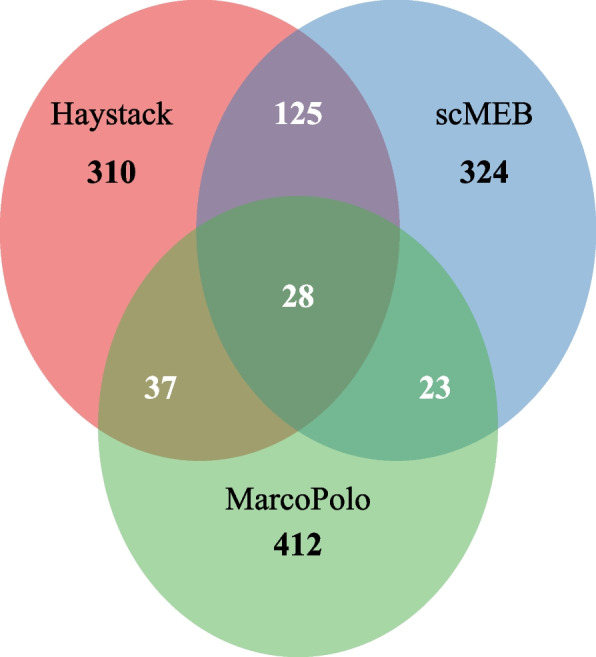
The common and unique genes of the most significant 500 DEGs detected by scMEB, Haystack, and MarcoPolo for the hESC dataset

**Fig. 3:**
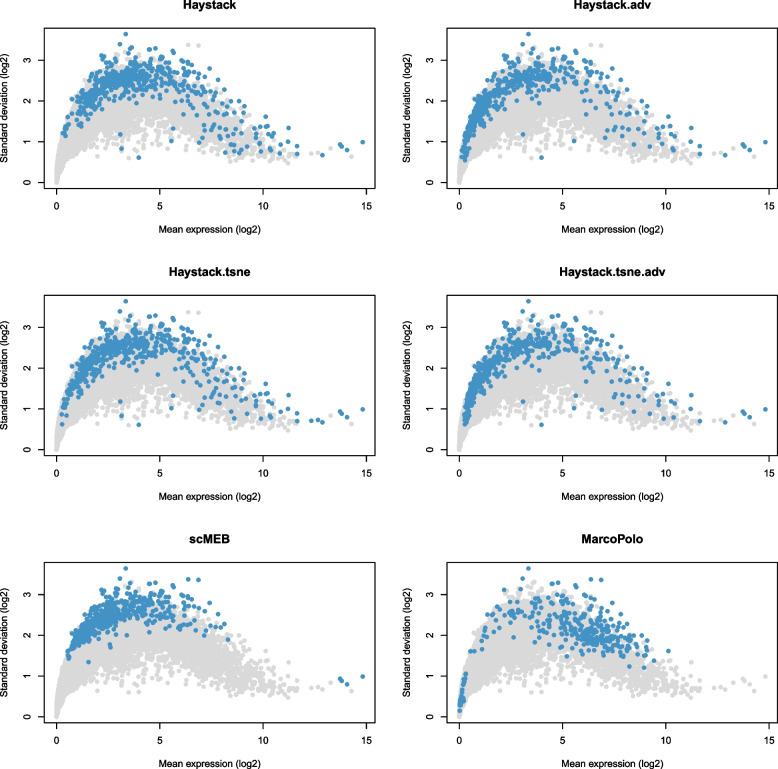
Scatter plot showing mean expression and standard deviation of each gene across single cells. Each point represents a gene, with blue points representing DEGs identified in the hESC dataset and gray points representing other genes

**Fig. 4:**
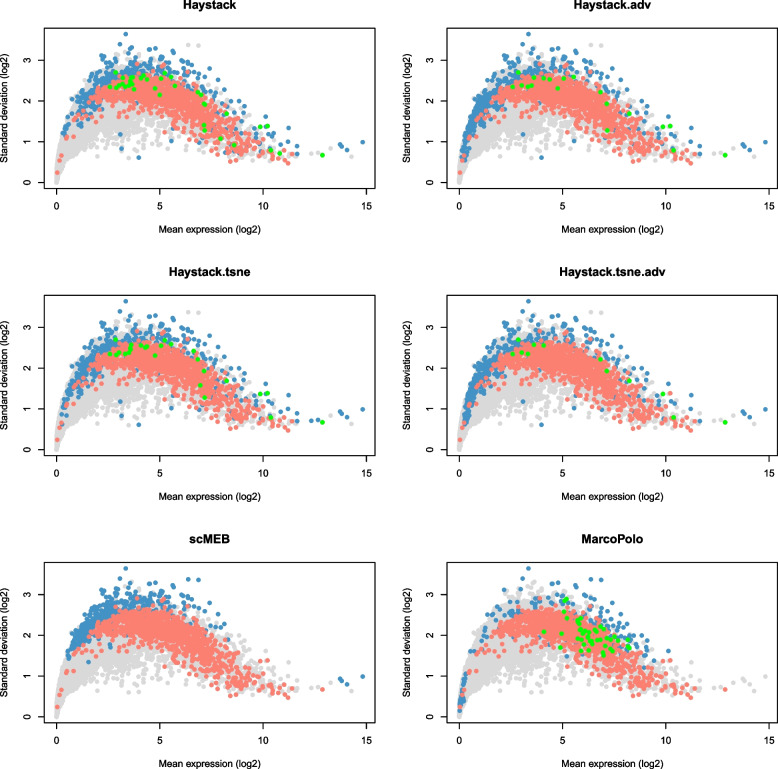
Scatter plot showing mean expression and standard deviation of each gene across single cells. Each point represents a gene, with blue points representing DEGs identified in the hESC dataset, red points representing SEGs, green points representing genes at the intersection of DEGs and SEGs, and gray points representing other genes

We derived lists of common and unique genes for the most significant 500 DEGs detected by three methods in the human embryogenic stem cell (hESC) dataset [19]. We found that scMEB and Haystack had more genes in common with each other than with MarcoPolo, suggesting that there are similarities in the results of singleCellHaystack and scMEB (Fig. 2). Additionally, we found a minor difference between the DEGs detected by the four modes of singleCellHaystack, and the average expressions of those DEGs almost covered the entire scope (Fig. 3). However, the DEGs detected by MarcoPolo and scMEB distributed more concentrated, both methods tended to select DEGs with higher standard deviations. DEGs detected by scMEB had a lower average expression, whereas the average expressions of DEGs detected by MarcoPolo were much higher. To understand the relationships between the detected DEGs and SEGs, we calculated the intersection of detected DEGs and SEGs. The SEGs used in scMEB were obtained through the total human SEGs excluding those that were not included in the hESC dataset. Figure 4 showed the distributions of SEGs and detected DEGs, as well as the intersection of these two types of genes. scMEB had the least number of overlapped genes, which demonstrated that it is reasonable to build a model with SEGs. Using Kumar’s [20] mouse dataset, we followed the same steps used for the hESC dataset and found a similar pattern to the human dataset (Supplementary file 1 Figs. S1–S3). Supplementary files 2–3 listed the SEGs and the most significant 500 DEGs detected by each method from the hESC data and Kumar data, as well as the differential statistics, including *p*-values, signed distances, and ranks. The four modes of singleCellHaystack, scMEB, and MarcoPolo returned the *p*-values, signed distances, and ranks for each gene, respectively. In addition, we got the number of SEGs that were included in the most significant 100, 200, 500, and 1,000 DEGs detected by each method (Supplementary file 1 Tables S2–S3). For the above two datasets, the scMEB had the smallest number of SEGs in the detected DEGs, which implied the smallest false discovery rate.

To validate the fidelity of DEGs identified by scMEB, we selected the most significant 100 DEGs detected by three methods from a human scRNA-seq data (hESC data). We then excluded the overlapping genes and analyzed the rest of the 74 DEGs detected by scMEB. We retrieved the biological function of these genes from the NCBI [21] website, and found that there were 71 genes related to human illness or embryonic development. Particularly, scMEB could detect a few key genes among the top 100 DEGs. We found that ‘ENSG00000136698’ is involved in signaling during embryonic development, and mutations in this gene may cause autosomal visceral heterotaxy and congenital heart disease; the cadherins encoded by ‘ENSG00000113361’ are essential for cell differentiation and morphogenesis, decreased expression of this gene may be related to tumor growth and metastasis; and ‘ENSG00000122691’ is important for embryonic development, and mutations in this gene would result in Saethre-Chotzen syndrome. Also, using the same procedures, we examined the DEGs detected by scMEB in a mouse dataset (Kumar data). Among the 80 DEGs uniquely detected by scMEB, 61 were specifically associated with mouse disease and development. More details about the functions of uniquely detected DEGs for two datasets could be accessed in Supplementary files 4–5.

### Comparison of identifying marker genes

We evaluated the performance of scMEB to identify marker genes. Cell markers are widely used for labeling cell populations and exploring cell compositions [22]. Following the two methods described by Kim et al. [15], we obtained marker genes and compared the performance of marker gene identification through the receiver operating characteristic (ROC) curve and area under the curve (AUC). The “The method to obtain marker genes” section introduces the details about the obtainment of marker genes.

**Fig. 5:**
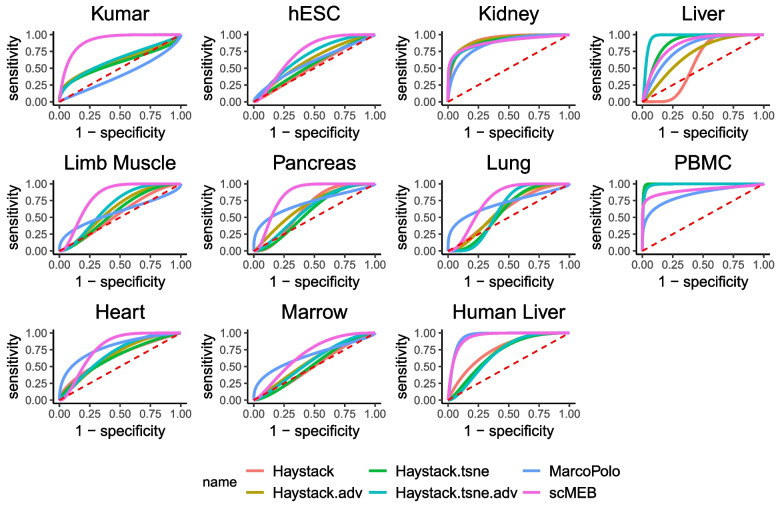
The ROC curves of singleCellHaystack, scMEB, and MarcoPolo for identifying 100 marker genes using 11 real datasets. The marker genes were selected using the maximum log fold change values, and the singleCellHaystack was carried out through four modes

We compared the ROC curves and AUC values of each method for marker gene identification using 11 real datasets (Fig. 5 and Supplementary file 1 Tables S4–S8). We first selected the top 100 genes with the maximum log fold change values for each dataset as marker genes. scMEB performed better than the other two methods for seven datasets, which accounted for $64\%$ of 11 datasets. Because there were minor differences in the AUC values between the four modes of singleCellHaystack, we chose the highest AUC value of the four modes as the value of singleCellHaystack. By doing so, this method performed the best for three out of 11 datasets. MarcoPolo showed the best performance for only one dataset. To evaluate the performance for different numbers of marker genes, we compared the AUC values of six methods in the 11 real datasets for the top 200, 300, 400, and 500 marker genes. Overall, scMEB performed the best in 7 or 8 of 11 datasets for the different numbers of selected marker genes, which coincided with the conclusion about the top 100 marker genes (Supplementary file 1 Tables S5–S8).

To reduce the influence of a specific calculation method on the marker genes, we retrieved a list of marker genes from the two databases. Only nine datasets corresponded to the tissues in the database and had enough marker genes for subsequent identification. Supplementary file 1 Table S9 shows the AUC values of each method for identifying marker genes. scMEB performed the best for five out of nine datasets, and singleCellHaystack had the best performance for the remaining four datasets. Therefore, we can draw similar conclusions about marker gene identification for these two methods of defining marker genes. We also found that MarcoPolo performed differently compared to the results in [15], despite conducting the same study using the same dataset. It is possible that MarcoPolo only used the genes with average expression levels in the top 30th percentile for data preprocessing, causing some DEGs to be removed before further analysis.

### Clustering analysis of DEGs

To characterize the performance of discriminating different cell types, we compared the clustering results of lists of DEGs detected by three types of methods and highly variable genes (HVGs) obtained by the *getTopHVGs()* function in the scran [13] package. We selected a data from the human and mouse datasets separately for clustering. For more details about the pre-processing of clustering and evaluation criteria, please refer to the “Clustering process and evaluation criteria” section.

**Fig. 6:**
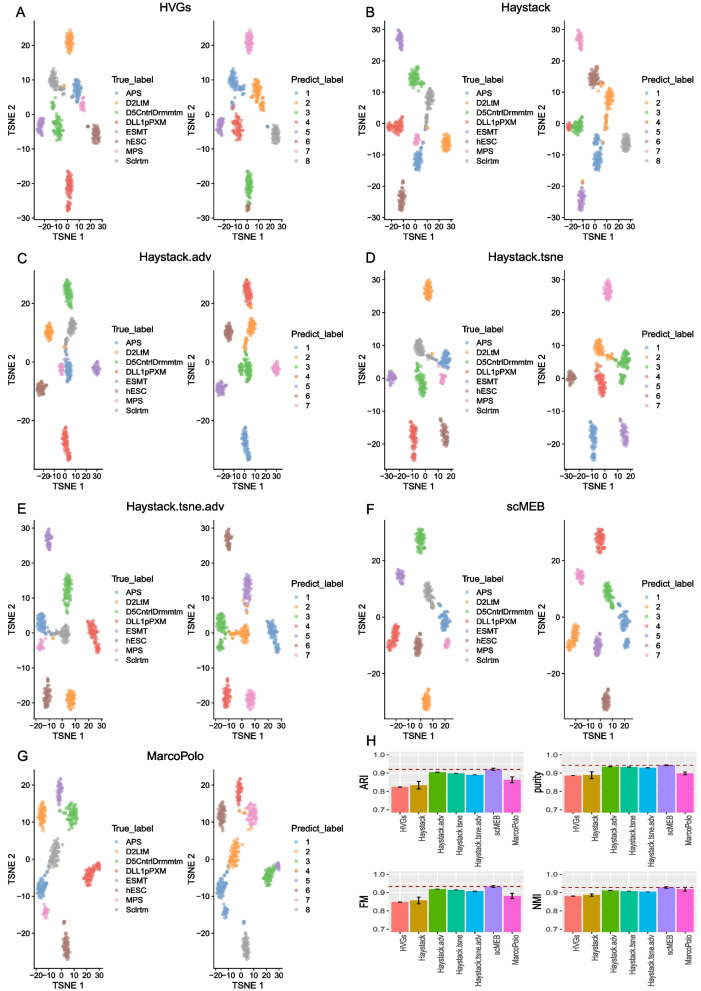
The clustering results of HVGs and DEGs detected by each method in a human (hESC data) dataset. **A**-**G** The t-SNE plots generated from hESC data using **A** HVGs as well as DEGs detected by **B** Haystack, **C** Haystack.adv, **D** Haystack.tsne, **E** Haystack.tsne.adv, **F** scMEB, and **G** MarcoPolo. The cells in the left panel are colored by predefined labels, and the cells in the right panel are colored by predicted labels. (H) Bar plots of comparison between clustering and predefined cell class labels using four clustering metrics. The red dashed line is the average value of scMEB for each metric

We first investigated the t-SNE plots generated from a human dataset (hESC data) using HVGs or DEGs detected by each method (Fig. 6 A–G). We found that HVGs and DEGs showed similar performance in separating different types of cells. Although DEGs detected by Haystack and MarcoPolo resulted in the same number of clustering types with true labels, all methods failed to distinguish the MPS from the APS cell types. The 20 average clustering results of the four metrics suggested that DEGs were superior to HVGs at separating cell types (Fig. 6 H). Of the DEGs, scMEB performed the best. Supplementary file 1 Fig. S4 shows the clustering results generated from a mouse dataset (Kumar data). Both HVGs and DEGs showed a clear separation for different cell types; however, a few cells were falsely clustered by HVGs and DEGs detected by singleCellHaystack and MarcoPolo, which were marked by red circles in the t-SNE plots. scMEB outperformed the other methods for the four clustering metrics.

### The comparison of gene ontology analysis

To further compare the performance of these methods, we explored the biological functions of detected DEGs by performing gene ontology (GO) enrichment analysis for the hESC dataset. We conducted GO enrichment analysis by selecting the most significant 500 DEGs detected by each method and mapping each of them to the ontologies in the GO database. Then, we compared the statistical significance of the top enriched ontologies. Refer to the “Gene ontology enrichment analysis” section for more details.

**Fig. 7:**
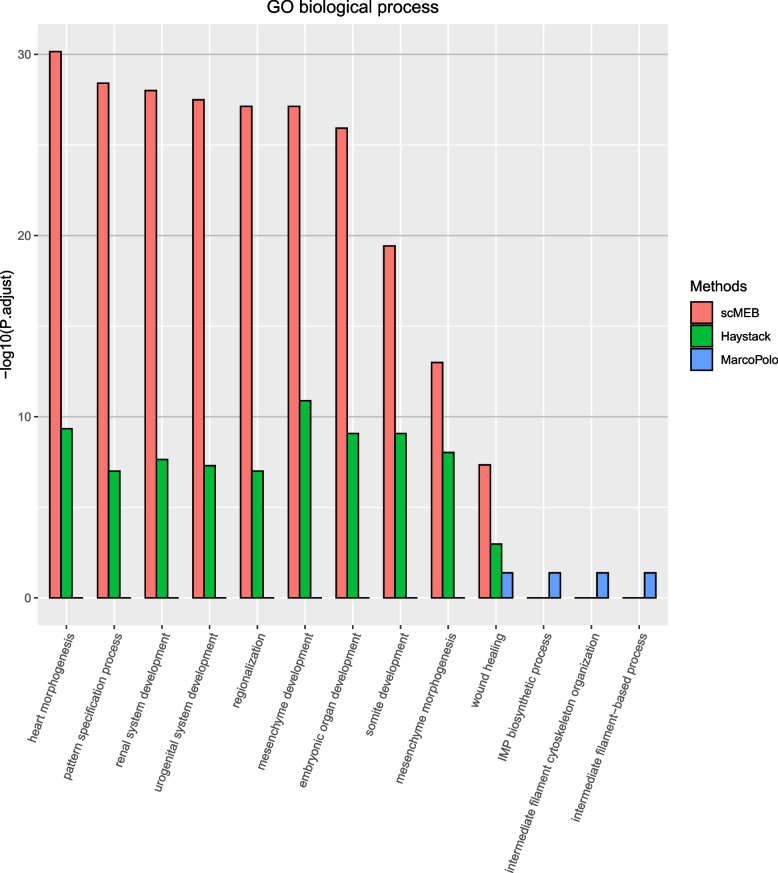
GO enrichment analysis of detected DEGs mapped to biological processes

For each functional category of GO, we combined the top five enriched GO terms for the three methods for comparison. For simplicity, we only compared the scMEB with Haystack and MarcoPolo. Figure 7 showed that the top five enriched terms for scMEB and Haystack were almost the same, and both differed from the MarcoPolo terms. We found the common top five enriched terms from scMEB and Haystack that described the development of different tissues in humans. These indicated that detected DEGs serve essential cellular functions, which also coincided with the source of the hESC dataset that originated from human embryonic stem cells at various stages of differentiation. The greater the $-log_{10}p.adjuct$, the more DEGs were mapped to this GO term, which was also called that DEGs were significantly enriched in this GO term. Therefore, compared with the other two methods, there were more genes in the 500 DEGs detected by scMEB mapped to these combined top five GO terms. Additionally, the DEGs detected by scMEB were significantly enriched in molecular function and cellular components (Supplementary file 1 Figs. S5 and S6). Nevertheless, the DEGs detected by MarcoPolo were less enriched for most GO terms. Supplementary file 6 contains the GO enrichment analysis results of three methods. From the outputs of three methods for GO enrichment analysis, the DEGs detected by scMEB were significantly enriched in 1,108 GO terms, the second was the Haystack (664 GO terms), and MarcoPolo had the least number of significantly enriched GO terms (29 GO terms). The number of DEGs detected by MarcoPolo that mapped to the database was also less than the numbers of scMEB and Haystack. The expressions of genes that were included in the same GO term may have some similarities or connections, causing these genes distributed more concentrated in the feature space. From the conclusions of Fig. 3, scMEB tended to detect DEGs that distributed more concentrated, however, the DEGs detected by Haystack distributed more dispersively. Therefore, the different distribution characteristics of detected DEGs led to a very different pattern of scMEB than the other two methods in the GO molecular function.

### Runtime comparison

**Fig. 8:**
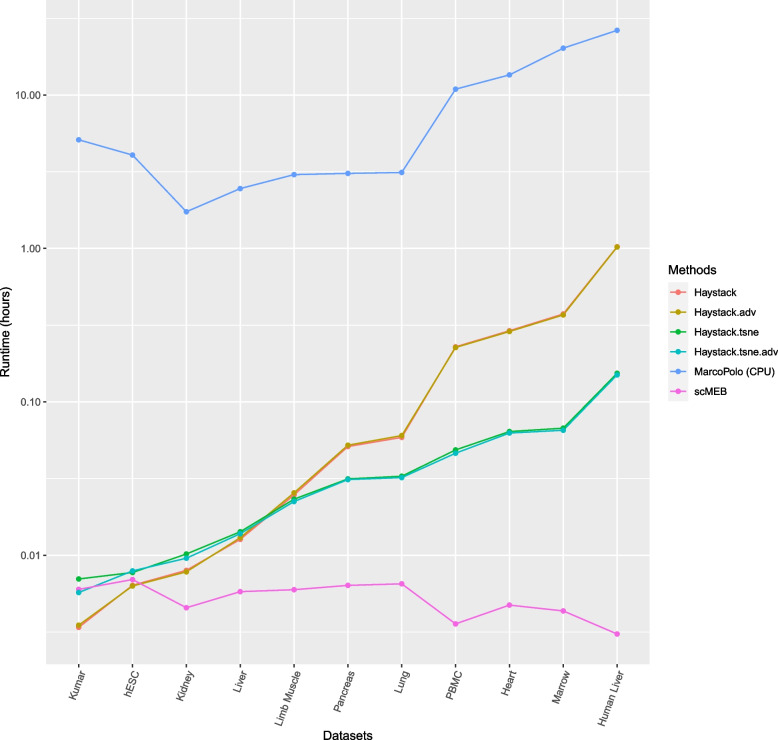
The runtime of different methods for 11 real datasets of various genes and sizes

We measured the runtime of the three methods by running the model for 11 real datasets. We only used the CPU version of MarcoPolo for comparison due to hardware restrictions. scMEB finished in seconds, singleCellHaystack in minutes, and MarcoPolo (CPU version) in hours (Fig. 8). MarcoPolo can involve fitting a mixture model for each gene, which means it takes a long time to estimate the parameters. Because MarcoPolo (GPU version) is $60\sim 90$ times faster than MarcoPolo (CPU version) [15], it is reasonable to infer that MarcoPolo (GPU version) would finish processing these 11 real datasets in minutes. The runtime of Haystack and Haystack.adv was close, and Haystack.tsne and Haystack.tsne.adv also had approach run times, which may be related to the type of inputs, as the former two methods used the first 50 PCs as inputs, while the latter two methods used two t-SNE coordinates. Inputting t-SNE coordinates for singleCellHaystack is faster than inputting 50 PCs, especially for a sizable dataset. scMEB was the fastest among all methods for 9 out of 11 datasets, and scMEB had a runtime similar to that of singleCellHaystack for the other two datasets. The details of runtime for these methods are shown in Supplementary file 1 Table S10.

## Discussion

scMEB is a fast method for detecting DEGs without clustering results. It finds an MEB based on a small part of known non-DEGs in the feature space. The DEGs are detected according to the distance from a mapped gene to the center of the sphere. Because it regards each gene as a vector, we were able to build the model without clustering the cell results, which reduced the effects of clustering results on DEG detection. Additionally, we only used a part of the genes and the first 50 PCs as inputs, which improved the calculation efficiency enormously. Sampling a portion of important cells before PCA could further accelerate the computation, which is very useful for high-throughput scRNA-seq data.

The differences in the expression distributions of DEGs and SEGs imply that building a model using a list of SEGs is reasonable. In addition, scMEB had a better or comparable performance for detecting marker genes compared to singleCellHasytack and MarcoPolo. The clustering results showed that DEGs detected by scMEB had a better performance in separating different cell types. Additionally, the GO enrichment analysis of detected DEGs suggested that scMEB has the potential to find more significant enriched genes than the other methods. We found that scMEB is similar to singleCellHaystack in terms of the number of common DEGs and the biological functions of the top enriched genes of the detected DEGs. Both methods performed better at marker gene detection than MarcoPolo. The comparison of runtimes for 11 real datasets with various genes and sizes coincided with the conclusion that scMEB is a fast method for scRNA-seq data differential expression analysis.

The discriminant function of scMEB is a dichotomic separation, which is different from the outputs of singleCellHaystack and MarcoPolo. singleCellHaystack provides *p*-values for each gene, and MarcoPolo returns a rank for each gene based on three criteria. Thus, either could be used to select a certain number of the most significant DEGs by setting a threshold. From this perspective, scMEB also provides a metric for ranking the genes, that is, the distance between the gene and the center of the ball in the feature space. The further the distance, the more significant the gene.

## Conclusions

We proposed a fast and clustering-independent scMEB method for DEG detection in scRNA-seq data. The analysis of 11 real datasets demonstrated that scMEB provides a new way to identify marker genes and predict genes with biological functions. Additionally, the selected DEGs could further improve the performance of clustering. Although scMEB has fast calculation, it suffers from a large number of genes because of the PCA step. We will consider this issue in our future work.

## Methods

### Processing of scRNA-seq data

We followed the steps by Vandenbon and Diez [14] to preprocess the datasets. First, we used counts per million to normalize the data. Then, we divided the expressions of each gene across the cells into detected and not detected and determined the threshold based on the median of the normalized data. Lastly, we kept the data for which genes were detected in more than 10 cells as well as cells with more than 100 detected genes for subsequent analysis.

### scRNA-seq datasets

We evaluated the performance of the scMEB by conducting analyses on 11 real scRNA-seq dataset, and comparing the results with singleCellHaystack and MarcoPolo. singleCellHaystack includes four modes ranging from simple to advanced, and uses the first 50 PCs or 2D t-SNE [23] coordinates as the input. For simplicity, we denoted the four modes as Haystack, Haystack.adv, Haystack.tsne, and Haystack.tsne.adv. Table 1 shows a brief summary of the 11 real datasets. The scRNA-seq data included different human and mouse tissues, and the number of cells varied from 200 to 8,000.

**Table 1:** Summary of 11 scRNA-seq datasets used for differentially expressed gene identification

Datasets	Organism	Cells (n)	Genes (n)	Classes (n)	Publication
hESC	Human	446	48981	8	[19]
PBMC	Human	3994	15716	8	[24]
Human Liver	Human	8444	20007	11	[25]
Kumar	Mouse	246	45159	3	[20]
Kidney	Mouse	519	23341	5	[26]
Liver	Mouse	714	23341	5	[26]
Limb Muscle	Mouse	1090	23341	6	[26]
Pancreas	Mouse	1564	23341	9	[26]
Lung	Mouse	1716	23341	11	[26]
Heart	Mouse	4365	23341	7	[26]
Marrow	Mouse	5037	23341	22	[26]

hESC: human embryogenic stem cell;

PBMC: peripheral blood mononuclear cell

### The framework for detecting DEGs in scRNA-seq datasets

The scMEB method extends the SFMEB [16] method, which has been previously used to identify DEGs in RNA-seq data, to scRNA-seq data. scMEB regards each gene as a point positioned in a feature space and builds a model with a small set of non-DEGs. The basic idea of scMEB is to find the smallest sphere $B(\varvec{c},R)$ to enclose the non-DEGs. Therefore, those genes outside the sphere, which are obviously different from the training data, could be identified as DEGs.

Using vector $\varvec{x}$ for a gene, a list of non-DE genes is represented by $\{\varvec{x}_i, i=1,2,\cdots ,n\}$. We formulate the model as follows

1 $$\begin{aligned} \begin{array}{rl} \underset{\varvec{c}, R, \xi }{min} &{}\quad R^2+C\sum _{i=1}^n\xi _{i}, \\ subjuct\;to &{}\quad ||\phi {(\varvec{x}_{i})}-\varvec{c}||^2\le R^2 +\xi _{i},\;\\ &{}\quad \xi _{i}\ge 0, \; i=1, 2, \cdots , n, \end{array} \end{aligned}$$

where $\xi$ is the slack variable, which is used to allow some points outside the sphere; $\varvec{c}$ and *R* are the center and radius of a sphere, respectively; *C* is a tuning parameter to control the radius and the number of errors; and $\phi$ is an unknown mapping that transforms the original vector into a high-dimensional vector in the feature space.

By solving the dual problem

2 $$\begin{aligned} \begin{array}{rl} \qquad \;\underset{\alpha }{min}\ {} &{}\quad \sum _{i=1}^{n}\sum _{j=1}^{n}\alpha _i\alpha _j\phi (\varvec{x}_i)^T\phi (\varvec{x}_j)-\sum _{i=1}^{n}\alpha _i\phi (\varvec{x}_i)^T\phi (\varvec{x}_j) \\ subject\;to &{} \quad \sum _{i=1}^{n}\alpha _i=1, \\ &{}\quad \;\, 0\le \alpha _i\le C, \; i=1,2,\cdots , n, \end{array} \end{aligned}$$

we obtain the decision function $f(\varvec{x}_i)=\left\| \phi {(\varvec{x}_{i})}-\hat{\varvec{c}}\right\| ^2-\hat{R}^2$. Because the formula is only related to the product of the mapped values, we replaced the product with a kernel function $K(\varvec{x}_i,\varvec{x}_j)$. There are multiple kernel functions to choose from; we used the radial basis function kernel ($K(\varvec{x}_i,\varvec{x}_j)=exp(-\nu \left\| \varvec{x}_i-\varvec{x}_j\right\| ^2)$) in our work. Therefore, the discriminant rule for detecting DEGs is

$$\begin{aligned} \left\{ \begin{array}{cc} \text {DE genes} &{}f(\varvec{x}_i)\!\!>\!\!0, \\ \text {non-DE genes} &{}otherwise. \end{array}\right. \end{aligned}$$

Since each gene is regarded as a vector in scMEB, the length of expressions of a gene across cells is the dimension of the gene. The greater the number of cells, the higher the gene dimensions. In reality, different types of data could be used as inputs in scMEB. Thus, we could use PCs as inputs to reduce the dimensions of the data. In the real data analysis, we used the first 50 PCs as inputs for scMEB. Replacing the original expression levels with the orthogonal PCs not only reduces the data noise, but also may improve the ability of scMEB to detect DEGs. Sampling important cells before PCA could further speed up the calculation. The threshold of the number of sampling cells could be set manually considering that some cells provide limited information for DE analysis, especially for cells with few detected genes. In the “Discussions about the settings in scMEB” section, we evaluated the performance of scMEB for different parameter settings in sampling, and found that sampling $10\%$ of all cells is a good choice for a big dataset. In our work, if the number of cells was more than 1,000, we followed the same sample rule as the advanced mode of singleCellHaystack [14] and randomly sampled 1,000 important cells from the total number of cells. The probability of each cell being selected was related to the number of detected genes in this cell. The more detected genes in the cell, the more likely the cell was to be selected. Figure 1 shows the workflow of scMEB for detecting DEGs. In this way, scMEB runs much faster than the other methods. Additionally, we followed the parametric tuning process of SFMEB [16], that is, fixing a parameter ($C=0.1$) and selecting another parameter $\nu$ from (0, 1) using a grid search. Considering that the expressions of some non-DEGs could be regarded as differential expressions, the reject rate was set at $10\%$ to control the scale of the sphere.

### Relation to SFMEB

There are mainly two extensions from the SFMEB to scMEB. First, different from the SFMEB which identifies DEGs under two biological conditions, scMEB could be extended to two or more potential types of cells or unknown labels datasets, which fits with the characteristic of scRNA-seq data. Second, instead of using the original expressions as inputs in SFMEB, scMEB incorporates PCA in the model, and uses the first 50 PCs as inputs, which improves the computation accuracy and speeds up the calculation enormously.

### Discussions about the settings in scMEB

We have conducted four analysis experiments to evaluate the performance of scMEB for different parameter settings in random sampling. Each analysis experiment was repeated 50 times and the AUC values were shown with boxplots. We selected data from the human (Human liver data) and mouse (Heart data) datasets separately, and the number of cells in these two datasets was over 4,000. We changed the sampled cells number to 100, 300, 500, 1,000, 1,500, and 2,000, the AUC values of scMEB increased greatly with the sampled number increased in the beginning, and gradually tended towards stability for a larger number of sampled cells (Supplementary file 1 Fig. S7 A–B). Meanwhile, the variability in AUC values reduced as the sampled number increased. Supplementary file 1 Fig. S7 C showed the AUC values of scMEB in seven datasets (cells number > 1,000) when sampled $2\%$, $5\%$, $10\%$, $15\%$, and $20\%$ of the total cells. We found that the AUC values changed minor when the sampled proportion was greater than or equal to $10\%$. At last, we sampled the same number of cells (1,000 cells) from each dataset, and showed the AUC values after running 50 times (Supplementary file 1 Fig. S7 D). The values for most of the datasets were stable, except for the Human liver data, in which AUC values changed comparatively greater than the other datasets. The reason may be that Human liver data has more total cells than the other datasets. Therefore, it is more reasonable to sample the important cells with a proportion rather than a fixed value.

We have conducted some analysis experiments to compare the performance and computational time when inputting original genes with sampling cells (sample + scMEB), inputting the top 50 PCs without sampling (PCA + scMEB), and inputting the top 50 PCs with sampling cells (Sample + PCA + scMEB) in eleven real datasets. Using the top 50 PCs as scMEB inputs would get higher AUC values than using the original genes for most datasets (Supplementary file 1 Fig. S8 A). There were minor differences in the median of AUC values between the sampling and without sampling, except that sampling cells would lead to a larger variance. In addition, compared with inputting the top 50 PCs, it would take a longer time when using original genes as inputs, and sampling cells before PCA could speed the calculation for a big data, especially for the Marrow and Human Liver datasets (Supplementary file 1 Fig. S8 B).

### The method to obtain marker genes

Kim et al. [15] provided two methods to obtain the marker genes. For the first method, we selected marker genes via the log fold change values. However, the log fold change is only related to two populations, and there may be more than two types of cells. We first grouped the cells for each gene by true labels. The original datasets provided the true labels of cells, which were obtained by fluorescence-activated cell sorting (FACS) technique or by manual curation based on the recognized markers. Next, We sorted the cells in ascending order based on the average expression of each group. The greatest difference in log average expression values between the consecutive cell types was regarded as the log fold change value of this gene. In this way, we selected the genes with maximum log fold change values as the marker genes. The second method for obtaining marker genes was to retrieve them from existing databases, such as the CellMarker [22] database and Panglao [27] database. Both databases provided a list of marker genes for different human and mouse tissues. We obtained the marker genes from those two databases that corresponded to the tissues of analyzed real datasets.

### Clustering process and evaluation criteria

To get the clustering results, we first selected 2,000 significant DEG or HVG genes and performed a principal component analysis (PCA) on the log-normalized expression values, and then we clustered the first 50 PCs using the *clusterCells()* function from scran [13]. We visualized the data with true labels and predicted labels using the t-SNE method. Four evaluation criteria were used to quantificationally evaluate the clustering results: adjusted rand index (ARI), purity, Fowlkes and Mallows (FM) index, and normalized mutual information (NMI).

### Gene ontology enrichment analysis

The GO database defines and describes the functions of genes and proteins and provides knowledge of the biological domain concerning biological processes, molecular functions, and cellular components [28]. We performed GO enrichment analysis by selecting some DEGs and mapping each of them to the ontologies in the GO database.

Each GO term is a pathway that includes a list of genes related to a certain biological function. The value that the number of genes in each pathway divides by the total number of genes for all the pathways is defined as ‘BgRatio’, which is only related to the GO database. If we map the detected DEGs to the pathways in the GO database, we have the total number of DEGs that could be mapped to GO database and the number of DEGs that mapped to each pathway. Then, we get another value for the ratio of these two numbers, which is called ‘GeneRatio’. The Fisher’s exact test or hypergeometric test is conducted to test the significance of the GeneRatio with respect to the BgRatio. The greater the -logPval, the more genes included in DEGs are enriched in this GO term.

## Supplementary Information

**Additional file 1.**

**Additional file 2.**

**Additional file 3.**

**Additional file 4.**

**Additional file 5.**

**Additional file 6.**

## Data Availability

The hESC, PBMC, and Kumar datasets were extracted from the R package DuoClustering2018 [29]; the Human Liver dataset was obtained from the R package HumanLiver [25]; and all mouse datasets were extracted from the Tabula Muris consortium data [26] that could be downloaded from the website https://tabula-muris.ds.czbiohub.org/.

## Abbreviations

scRNA-seq: Single-cell RNA sequencing
DEGs: Differentially expressed genes
SEGs: Stably expressed genes
HVGs: Highly variable genes
SFMEB: Scaling-free minimum enclosing ball
scMEB: Single-cell minimum enclosing ball
hESC: Human embryogenic stem cell
PBMC: Peripheral blood mononuclear cell
GO: Gene ontology
PCA: Principal component analysis
PCs: Principal components
ROC: Receiver operating characteristic
AUC: Area under the curve
ARI: Adjusted rand index
FM: Fowlkes and Mallows
NMI: Normalized mutual information
