# Supplementary material for: scMEB: a fast and clustering-independent method for detecting differentially expressed genes in single-cell RNA-seq data
Source: BMC Genomics. 2023 May 25;24:280. doi: 10.1186/s12864-023-09374-6 (PMC10210493; doi:10.1186/s12864-023-09374-6)
Supplement: Supplementary file 1 — Additional file 1. [file 12864_2023_9374_MOESM1_ESM.pdf]

Supplemental materials for “scMEB: A fast and clustering-independent method for detecting differentially expressed genes in single-cell RNA-seq data”

Jiadi Zhu [1] and Youlong Yang [1,*]

[1]*Department of Mathematics and Statistics, Xidian University, Xi’an, Shaanxi, China*

Supplementary Figures (8) and Table (10)

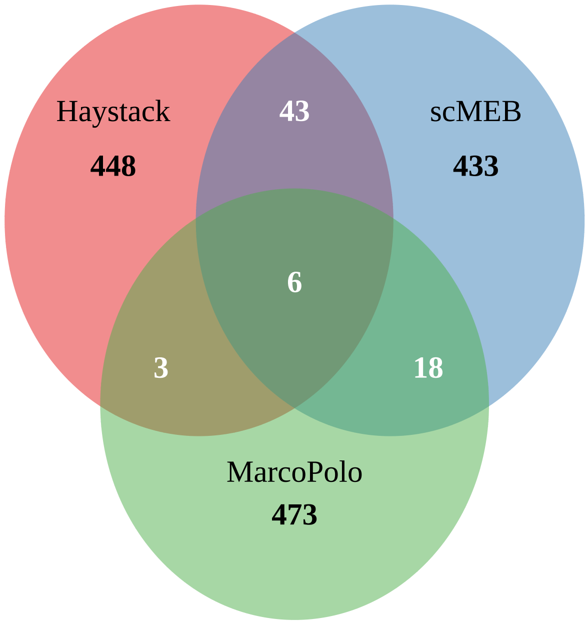

Figure S1: The common and unique genes of the most significant 500 DEGs detected by scMEB, Haystack, and MarcoPolo for the Kumar dataset.

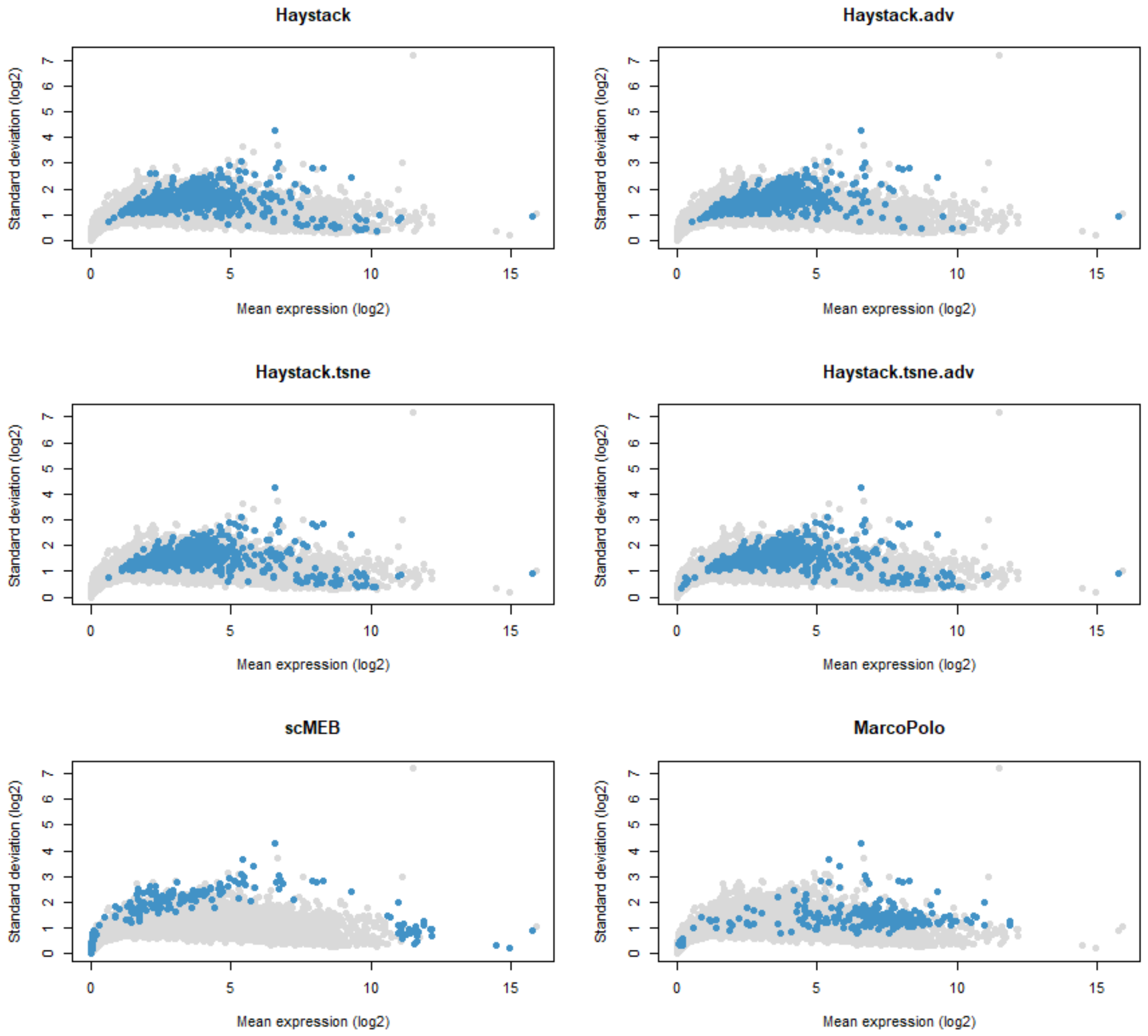

Figure S2: Scatter plot showing mean expression and standard deviation of each gene across single cells. Each point represents a gene, with blue points representing DEGs identified in the Kumar dataset and gray points representing other genes.

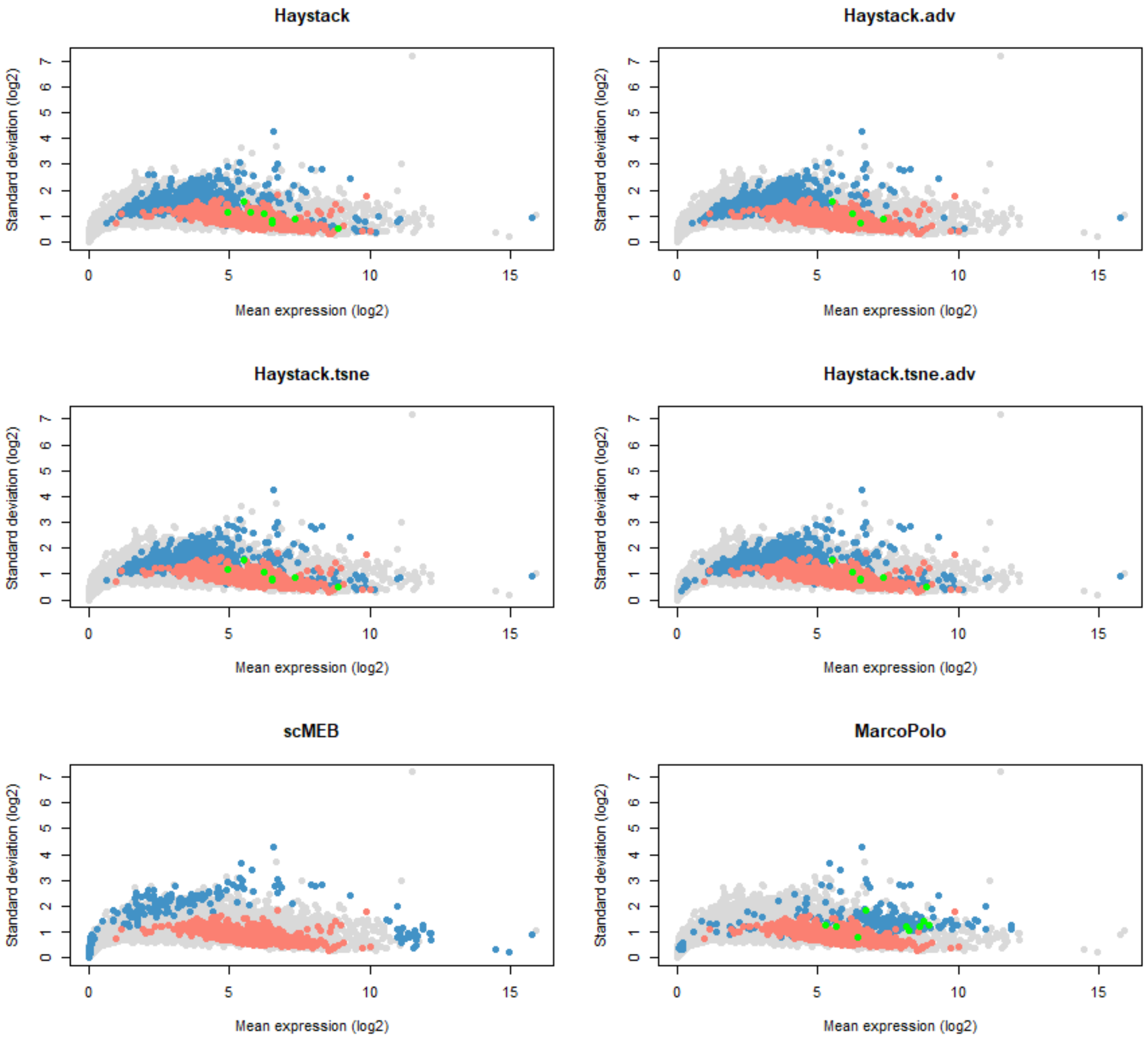

Figure S3: Scatter plot showing mean expression and standard deviation of each gene across single cells. Each point represents a gene, with blue points representing DEGs identified in the Kumar dataset, red points representing SEGs, green points representing genes at the intersection of DEGs and SEGs, and gray points representing other genes.

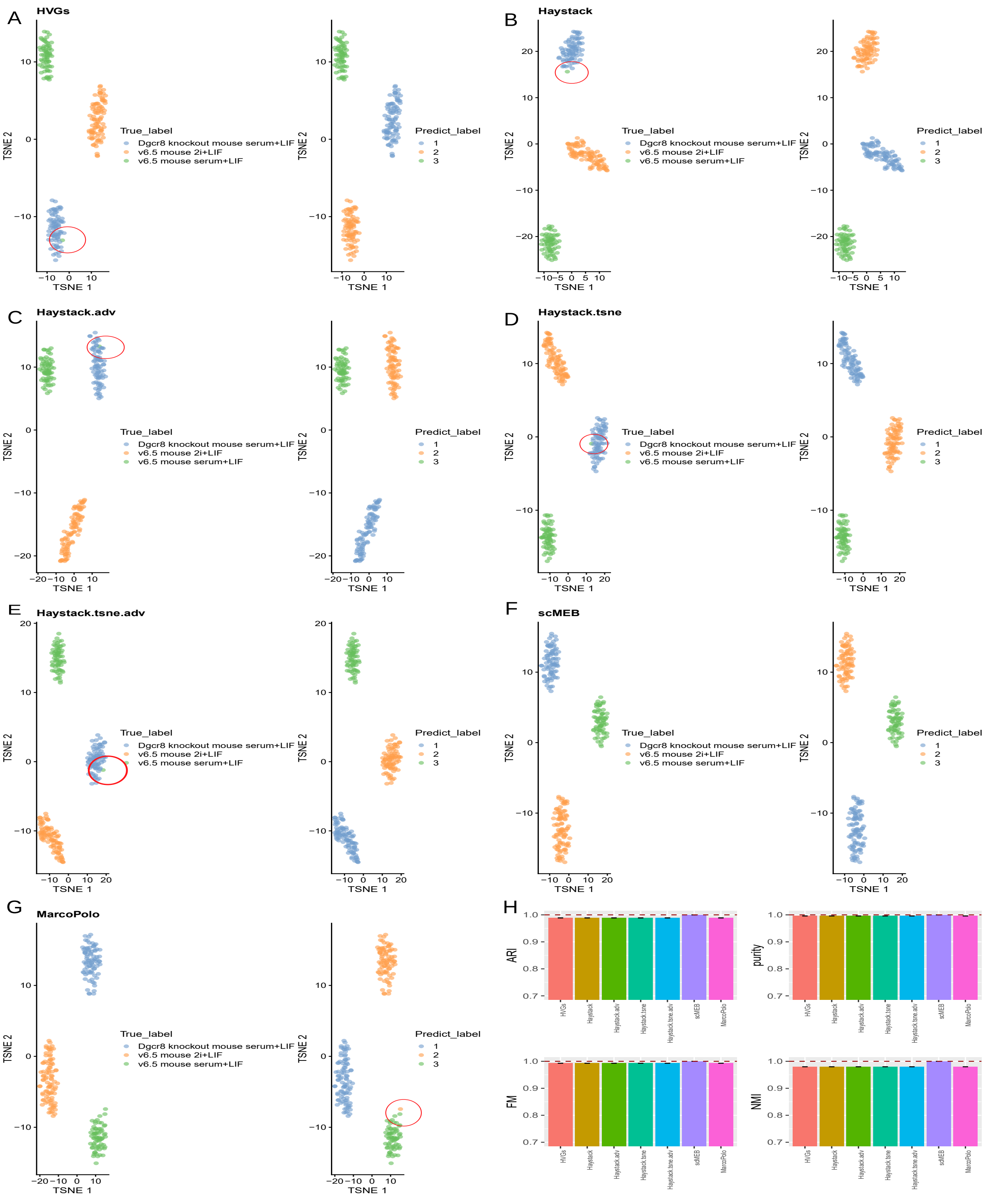

Figure S4: The clustering results of HVGs and DEGs detected by each method in a mouse dataset (Kumar data). (A-G) The t-SNE plots generated from Kumar data using (A) HVGs as well as DEGs detected by (B) Haystack, (C) Haystack.adv, (D) Haystack.tsne, (E) Haystack.tsne.adv, (F) scMEB, and (G) MarcoPolo. The cells in the left panel are colored by predefined labels, and the cells in the right panel are colored by predicted labels. The falsely clustered cells are marked by a red circle. (H) Bar plots of comparison between clustering and predefined cell class labels using four clustering metrics. The red dashed line is the average value of scMEB for each metric.

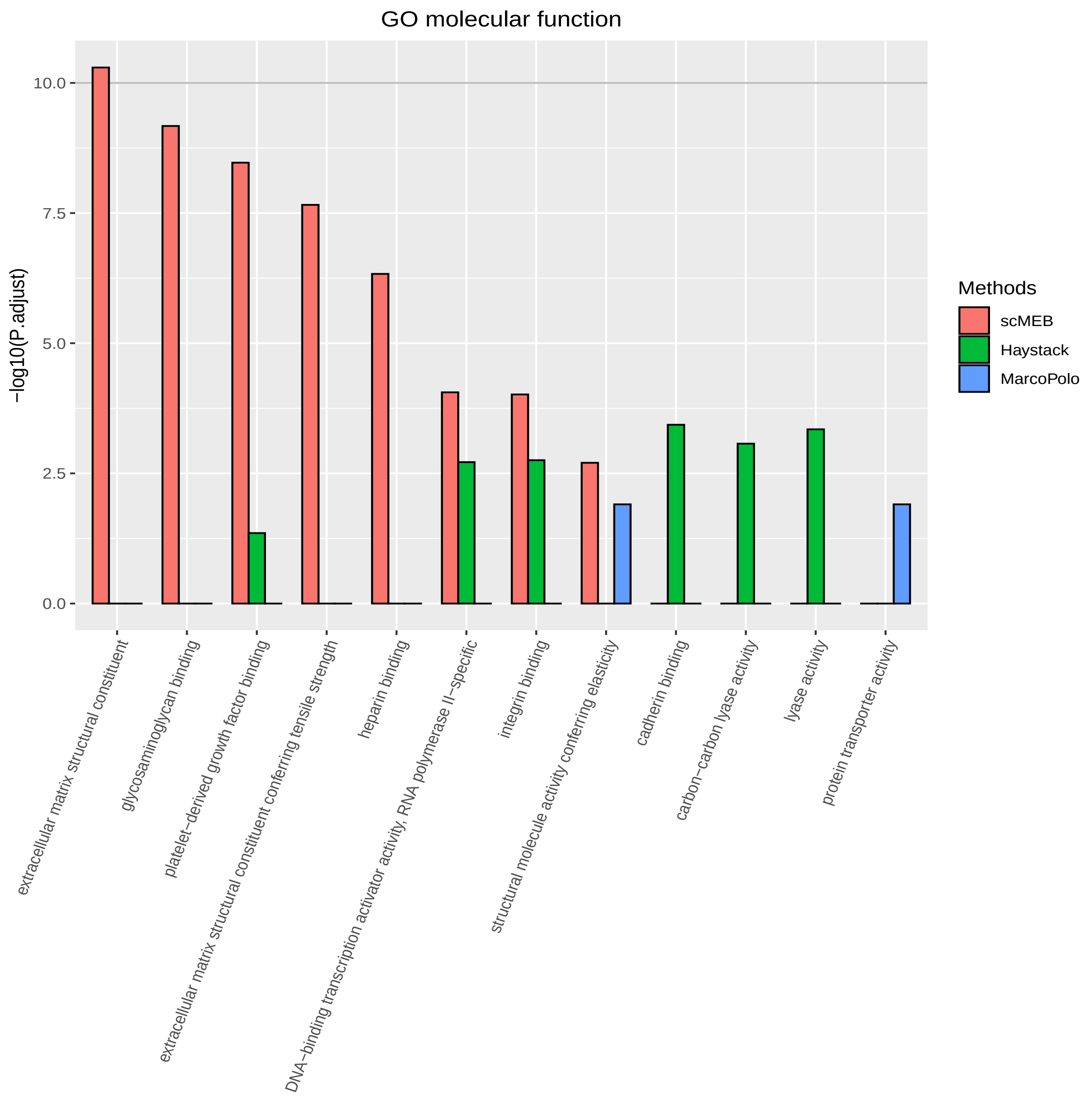

Figure S5: GO enrichment analysis of detected DEGs mapped to molecular functions.

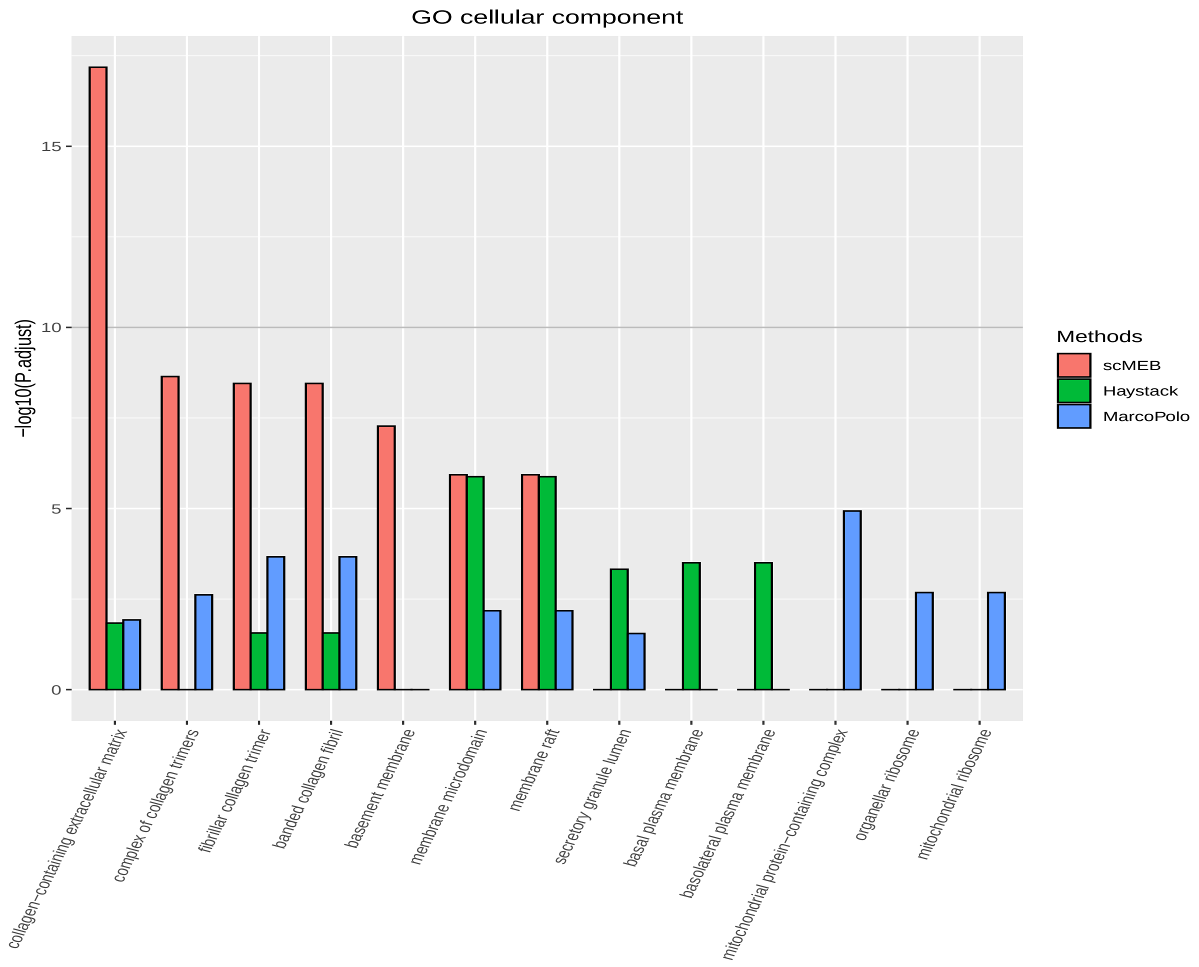

Figure S6: GO enrichment analysis of detected DEGs mapped to cellular components.

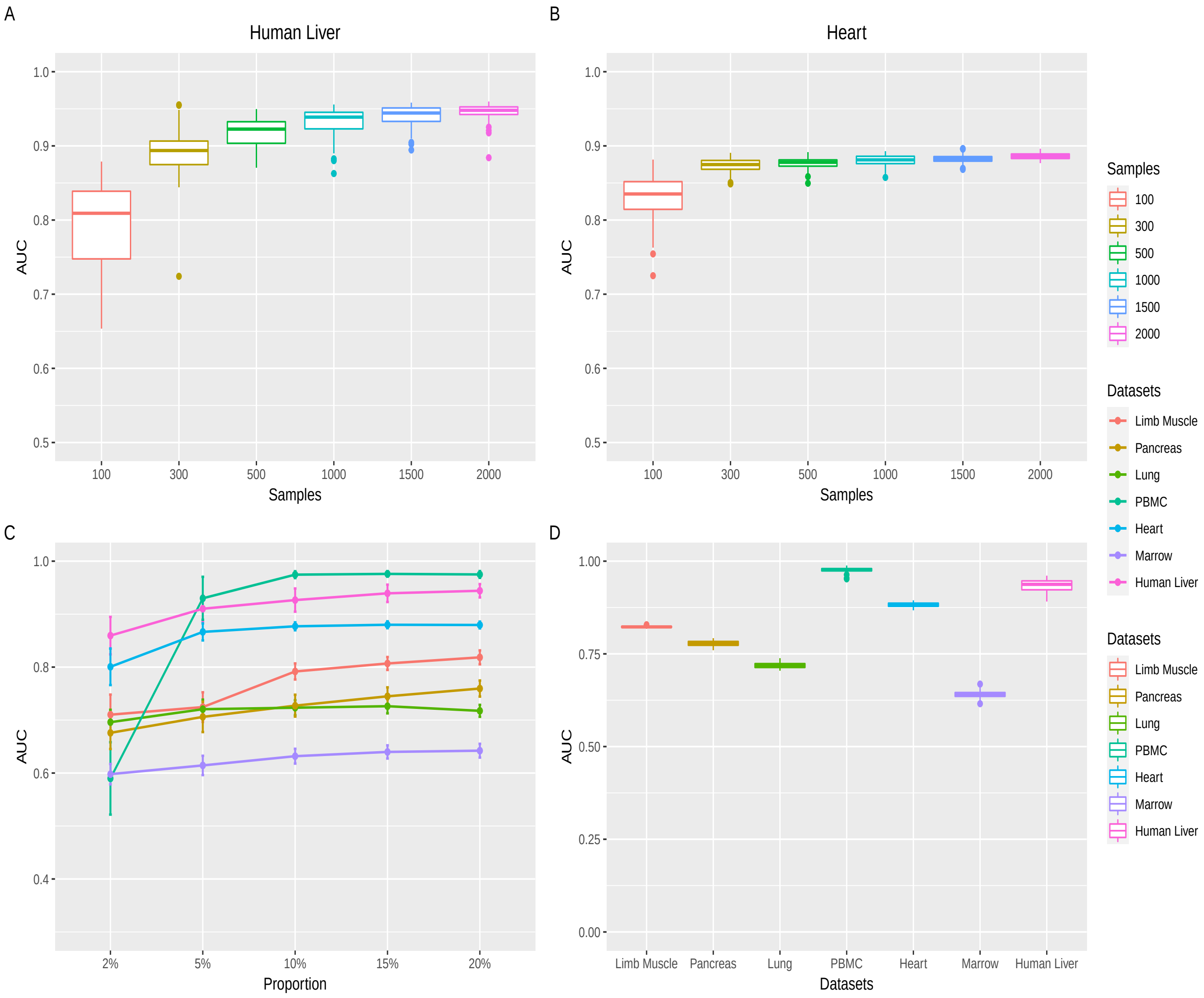

Figure S7: The AUC values of scMEB for different parameter settings in random sampling. (A) The boxplots of AUC values when sampled 100, 300, 500, 1,000, 1,500, and 2,000 cells from the Human Liver data. (B) The boxplots of AUC values when sampled 100, 300, 500, 1,000, 1,500, and 2,000 cells from the Heart data. (C) The line chart of AUC values when sampled 2%, 5%, 10%, 15%, and 20% of total cells from seven datasets. (D) The boxplots of AUC values when sampled the same number of 1,000 cells from seven datasets.

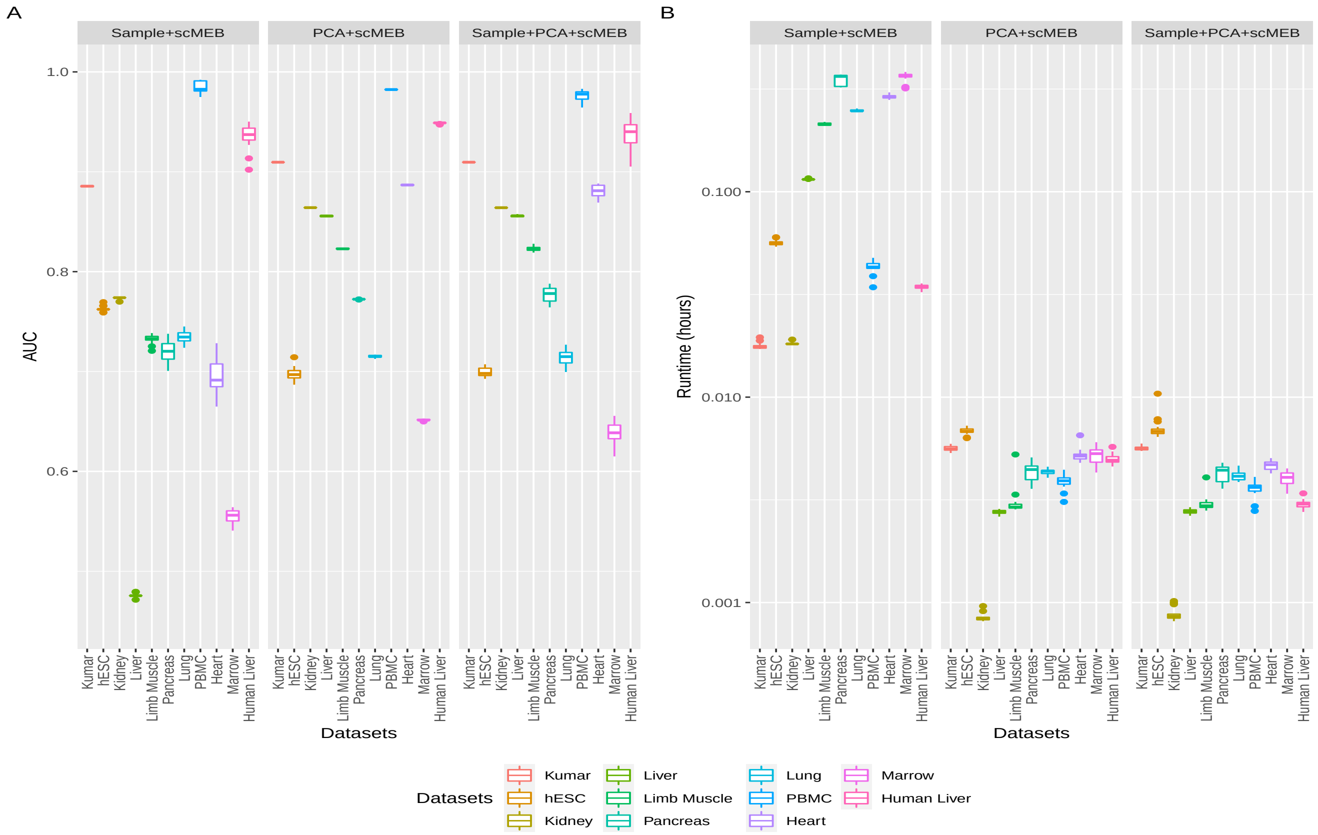

Figure S8: The performance and computational time of scMEB when using original genes with sampling cells (sample + scMEB), top 50 PCs without sampling (PCA + scMEB), or top 50 PCs and sampling cells (Sample + PCA + scMEB). (A) The boxplots of AUC values for 11 real datasets. (B) The boxplots of computational time for 11 real datasets.

Table S1: The comparison between the reference numbers of classes and clustering results obtained by the different parameter settings using the graph-based method from the scran package.

Datasets	Ref	Estimation										
		k=5	k=10	k=15	type= "number"	type= "jaccard"	walktrap	louvain	infomap	fast greedy	label prop	leading eigen
Kumar	3	3	3	3	3	3	3	3	3	3	3	3
hESC	8	10	8	7	9	9	8	7	9	6	8	8
Kidney	5	11	6	5	8	8	6	6	10	3	9	5
Liver	5	16	8	7	8	14	8	8	14	5	14	8
Limb Muscle	6	17	16	9	11	11	16	7	11	4	8	6
Pancreas	9	17	12	12	13	14	12	11	20	5	21	12
Lung	11	30	22	13	24	25	22	16	24	10	23	19
PBMC	8	21	16	15	11	16	16	8	19	5	9	8
Heart	7	28	20	19	19	19	20	12	32	7	24	14
Marrow	22	30	22	24	30	19	22	11	42	5	36	14
Human Liver	11	71	34	28	31	29	34	20	70	8	85	24

Table S2: For hESC data, the number of SEGs is included in the most significant 100, 200, 500, and 1,000 DEGs for the six methods.

Methods	100	200	500	1000
Haystack	5	10	37	79
Haystack.adv	2	5	19	62
Haystack.tsne	4	6	26	72
Haystack.tsne.adv	1	4	12	49
MarcoPolo	6	18	45	90
scMEB	0	0	0	0

Table S3: For Kumar data, the number of SEGs is included in the most significant 100, 200, 500, and 1,000 DEGs for the six methods.

Methods	100	200	500	1000
Haystack	1	1	8	17
Haystack.adv	1	3	4	12
Haystack.tsne	2	3	7	20
Haystack.tsne.adv	1	3	6	20
MarcoPolo	1	5	9	13
scMEB	0	0	0	0

Table S4: The AUC values of singleCellHaystack, scMEB, and MarcoPolo for identifying the top 100 marker genes using 11 real datasets. The marker genes were selected using the maximum log fold change values, and the singleCellHaystack was carried out through four modes. The highest AUC value for each dataset is highlighted in bold.

Datasets	Haystack	Haystack.adv	Haystack.tsne	Haystack.tsne.adv	MarcoPolo	scMEB
Kumar	0.6012	0.6198	0.5788	0.6306	0.4088	**0.9091**
hESC	0.5264	0.6175	0.5525	0.6762	0.5868	**0.7273**
Kidney	**0.9215**	0.9201	0.9005	0.8847	0.8577	0.8916
Liver	0.5883	0.7104	0.8876	**0.9670**	0.8042	0.8561
Limb Muscle	0.5414	0.6237	0.5801	0.6625	0.5804	**0.8050**
Pancreas	0.6321	0.6712	0.5823	0.6213	0.7175	**0.8406**
Lung	0.5978	0.6183	0.5874	0.6105	0.7078	**0.7607**
PBMC	0.9947	0.9936	**0.9949**	0.9889	0.8370	0.9729
Heart	0.6465	0.6585	0.6376	0.6823	0.7915	**0.7925**
Marrow	0.5035	0.5828	0.5237	0.5723	0.6636	**0.6823**
Human Liver	0.7509	0.7016	0.7010	0.6921	**0.9498**	0.9367

Table S5: The AUC values of singleCellHaystack, scMEB, and MarcoPolo for identifying the top 200 marker genes using 11 real datasets. The marker genes were selected using the maximum log fold change values, and the singleCellHaystack was carried out through four modes. The highest AUC value for each dataset is highlighted in bold.

Datasets	Haystack	Haystack.adv	Haystack.tsne	Haystack.tsne.adv	MarcoPolo	scMEB
Kumar	0.7048	0.7175	0.7002	0.7304	0.7229	**0.8620**
hESC	0.5401	0.6112	0.5456	0.6742	0.5744	**0.7187**
Kidney	0.6854	0.7231	0.7903	**0.8286**	0.6622	0.8255
Liver	0.5313	0.4799	0.4954	0.6882	0.6943	**0.7341**
Limb Muscle	0.5984	0.6751	0.6381	0.7008	0.6276	**0.8229**
Pancreas	0.5809	0.7148	0.5623	0.6739	0.6720	**0.8188**
Lung	0.6670	0.6901	0.6387	0.6556	0.7486	**0.7705**
PBMC	0.9942	0.9927	**0.9943**	0.9864	0.8333	0.8037
Heart	0.6278	0.6493	0.6093	0.6688	0.7665	**0.7797**
Marrow	0.5076	0.5918	0.5067	0.5847	0.6563	**0.6609**
Human Liver	0.8192	0.7729	0.7891	0.7708	**0.9482**	0.9162

Note: for the PBMC data, because the genes that satisfied the conditions of being marker genes are the same for the selection of 200, 300, 400, and 500 marker genes, the AUC values of each method are not changed.

Table S6: The AUC values of singleCellHaystack, scMEB, and MarcoPolo for identifying the top 300 marker genes using 11 real datasets. The marker genes were selected using the maximum log fold change values, and the singleCellHaystack was carried out through four modes. The highest AUC value for each dataset is highlighted in bold.

Datasets	Haystack	Haystack.adv	Haystack.tsne	Haystack.tsne.adv	MarcoPolo	scMEB
Kumar	0.7569	0.7665	0.7603	0.7844	0.7714	**0.8355**
hESC	0.5463	0.5949	0.5388	0.6661	0.5531	**0.7188**
Kidney	0.7130	0.7477	0.7988	**0.8366**	0.6789	0.8337
Liver	0.4895	0.5665	0.5154	0.7079	0.6880	**0.7718**
Limb Muscle	0.6207	0.6934	0.6587	0.7204	0.6313	**0.8172**
Pancreas	0.5709	0.7109	0.5525	0.6696	0.6547	**0.7970**
Lung	0.6718	0.6913	0.6463	0.6620	0.7356	**0.7663**
PBMC	0.9942	0.9927	**0.9943**	0.9864	0.8333	0.8037
Heart	0.6329	0.6582	0.6113	0.6736	0.7630	**0.7826**
Marrow	0.5146	0.5940	0.4939	0.5902	**0.6472**	0.6457
Human Liver	0.8594	0.8180	0.8334	0.8167	**0.9425**	0.8986

Table S7: The AUC values of singleCellHaystack, scMEB, and MarcoPolo for identifying the top 400 marker genes using 11 real datasets. The marker genes were selected using the maximum log fold change values, and the singleCellHaystack was carried out through four modes. The highest AUC value for each dataset is highlighted in bold.

Datasets	Haystack	Haystack.adv	Haystack.tsne	Haystack.tsne.adv	MarcoPolo	scMEB
Kumar	0.7789	0.7919	0.7826	0.8064	0.7893	**0.8261**
hESC	0.5441	0.5956	0.5382	0.6683	0.5455	**0.7244**
Kidney	0.7081	0.7436	0.7833	0.8270	0.6667	**0.8294**
Liver	0.4906	0.5780	0.5285	0.7093	0.6671	**0.7744**
Limb Muscle	0.6195	0.6882	0.6581	0.7200	0.6408	**0.8055**
Pancreas	0.5710	0.6969	0.5547	0.6599	0.6280	**0.7830**
Lung	0.6600	0.6771	0.6390	0.6520	0.7207	**0.7419**
PBMC	0.9942	0.9927	**0.9943**	0.9864	0.8333	0.8037
Heart	0.6034	0.6366	0.5835	0.6541	0.7425	**0.7713**
Marrow	0.5139	0.5940	0.4934	0.5898	0.6415	**0.6392**
Human Liver	0.8844	0.8515	0.8613	0.8495	**0.9384**	0.8781

Table S8: The AUC values of singleCellHaystack, scMEB, and MarcoPolo for identifying the top 500 marker genes using 11 real datasets. The marker genes were selected using the maximum log fold change values, and the singleCellHaystack was carried out through four modes. The highest AUC value for each dataset is highlighted in bold.

Datasets	Haystack	Haystack.adv	Haystack.tsne	Haystack.tsne.adv	MarcoPolo	scMEB
Kumar	0.7861	0.8005	0.7939	**0.8155**	0.8001	0.8128
hESC	0.5426	0.5932	0.5431	0.6694	0.5438	**0.7246**
Kidney	0.7066	0.7434	0.7755	0.8215	0.6625	**0.8250**
Liver	0.4907	0.5838	0.5341	0.7136	0.6600	**0.7694**
Limb Muscle	0.6304	0.6948	0.6675	0.7278	0.6508	**0.8072**
Pancreas	0.5621	0.6919	0.5414	0.6631	0.6205	**0.7638**
Lung	0.6546	0.6697	0.6391	0.6513	0.7025	**0.7194**
PBMC	0.9942	0.9927	**0.9943**	0.9864	0.8333	0.8037
Heart	0.5820	0.6220	0.5626	0.6400	0.7326	**0.7571**
Marrow	0.5157	0.6022	0.4918	0.5956	**0.6383**	0.6322
Human Liver	0.8990	0.8715	0.8791	0.8696	**0.9361**	0.8597

Table S9: The AUC values of singleCellHaystack, scMEB, and MarcoPolo for identifying 100 marker genes using 9 real datasets. The marker genes were retrieved from the CellMarker database and Panglao database, and the singleCellHaystack was carried out through four modes. The highest AUC value for each dataset is highlighted in bold.

Datasets	Haystack	Haystack.adv	Haystack.tsne	Haystack.tsne.adv	MarcoPolo	scMEB
Kidney	0.7552	**0.7642**	0.7474	0.7557	0.6799	0.7415
Liver	0.5782	0.4729	0.5289	0.6944	0.6630	**0.8535**
Limb Muscle	0.8213	0.8389	0.8190	0.8586	0.7775	**0.8707**
Pancreas	0.7528	0.8885	0.8132	0.8769	0.7837	**0.9252**
Lung	0.7494	**0.7561**	0.7404	0.7505	0.6766	0.7269
PBMC	0.7557	0.7852	0.7629	**0.7935**	0.6839	0.6080
Heart	0.8907	0.9015	0.8849	0.8951	0.8409	**0.9273**
Marrow	0.6276	0.6829	0.6174	0.6765	0.6914	**0.7504**
Human Liver	0.7785	0.7784	0.7719	**0.7860**	0.7171	0.6918

Table S10: The runtime of different methods for 11 real datasets of various genes and sizes (seconds). The shortest runtime for each dataset is highlighted in bold.

Datasets	No.cell	No.gene	Haystack	Haystack.adv	Haystack.tsne	Haystack.tsne.adv	MarcoPolo (CPU)	scMEB
Kumar	246	45159	**12.20**	12.61	25.23	20.59	18394.44	21.59
hESC	446	48981	22.94	**22.72**	27.81	28.48	14624.18	25.02
Kidney	519	23341	28.72	28.08	36.70	34.45	6246.59	**16.39**
Liver	714	23341	45.72	46.86	51.25	49.77	8843.62	**20.86**
Limb Muscle	1090	23341	89.11	91.94	83.59	80.69	10902.86	**21.48**
Pancreas	1564	23341	184.17	187.88	113.32	112.19	11106.97	**22.92**
Lung	1716	23341	211.31	217.11	117.94	115.58	11263.17	**23.46**
PBMC	3994	15716	822.17	814.25	175.05	166.58	39314.76	**7.69**
Heart	4365	23341	1046.73	1035.09	230.64	225.51	48700.78	**17.05**
Marrow	5037	23341	1346.53	1327.36	242.60	235.13	72734.55	**15.62**
Human Liver	8444	20007	3692.52	3679.70	553.00	539.73	95065.44	**11.05**
